# Risk Factors of Adverse Maternal Outcome among SARS-CoV-2 Infected Critically Ill Pregnant Women in Serbia

**DOI:** 10.3390/jcm12123902

**Published:** 2023-06-07

**Authors:** Sladjana Mihajlovic, Jelena Trifunovic Kubat, Dejan Nikolic, Milena Santric-Milicevic, Biljana Milicic, Nemanja Dimic, Milan Lackovic

**Affiliations:** 1 Department of Obstetrics and Gynecology, University Hospital “Dragisa Misovic”, 11000 Belgrade, Serbia; sladjana.mihajlovic@dragisamisovic.bg.ac.rs (S.M.);; 2 Faculty of Medicine, University of Belgrade, 11000 Belgrade, Serbia; dejan.nikolic@udk.bg.ac.rs (D.N.); milena.santric-milicevic@med.bg.ac.rs (M.S.-M.);; 3 Department of Physical Medicine and Rehabilitation, University Children’s Hospital, 11000 Belgrade, Serbia; 4 Institute of Social Medicine, Faculty of Medicine, University of Belgrade, 11000 Belgrade, Serbia; 5 Faculty of Medicine, School of Public Health and Health Management, University of Belgrade, 11000 Belgrade, Serbia; 6 Department of Medical Statistics and Informatics, School of Dental Medicine, University of Belgrade, 11000 Belgrade, Serbia; biljana.milicic@sbb.rs; 7 Clinic for Anesthesiology and Intensive Care, University Hospital “Dragisa Misovic”, 11000 Belgrade, Serbia

**Keywords:** pregnancy, COVID-19, mortality, comorbidities, treatment, multidisciplinary approach

## Abstract

Background and Objectives: During the COVID-19 pandemic, Serbia has faced devastating losses related to increased mortality rates among men and women of all ages. With 14 registered cases of maternal death in 2021, it became obvious that pregnant women are faced with a serious threat that jeopardises their life as well as the life of their unborn child. Studying the consequences of the COVID-19 pandemic on maternal outcomes is vivifying and stimulating for many professionals and decision-makers, and knowing the contextual characteristics can facilitate the application of literature findings in practice. Therefore, the aim of this study was to present findings of maternal mortality in Serbia associated with SARS-CoV-2 infected and critically ill pregnant women. Methods: Clinical status and pregnancy-related features were analysed for a series of 192 critically ill pregnant women with confirmed SARS-CoV-2 infection. According to the treatment outcome, pregnant women were divided in two study groups: a group of survivors and a group of deceased patients. Results: A lethal outcome was recorded in seven cases. Pregnant women in the deceased group were presenting at admission more commonly with X-ray–confirmed pneumonia, a body temperature of >38 °C, cough, dyspnea, and fatigue. They were more likely to have a progression of the disease, to be admitted to intensive care unit, and be dependent from mechanical ventilation, as well as to have nosocomial infection, pulmonary embolism, and postpartum haemorrhage. On average, they were in their early third trimester of pregnancy, presenting more commonly with gestational hypertension and preeclampsia. Conclusions: Initial clinical manifestations of SARS-CoV-2 infection, such as dyspnea, cough, fatigue, and fever, could be a potent factors in risk stratification and outcome prediction. Prolonged hospitalization, ICU admission, and associated risk of hospital-acquired infections require strict microbiological surveillance and should be a constant reminder of rational antibiotics use. Understanding and identification of risk factors associated with poor maternal outcomes among pregnant women infected with SARS-CoV-2 should warn medical professionals of potentially unwanted outcomes and can be used for organising an individualised treatment for a pregnant patient’s specific needs, including a guide to necessary consultations with medical specialists in various fields.

## 1. Introduction

Maternal mortality rates range widely, from one in six in the most undeveloped parts of the world, to one in 30,000 in the most progressive healthcare systems [1]. During the last few decades, the overall predominant cause leading to maternal deaths has changed; incidence of puerperal sepsis as a primary cause of maternal death has decreased, while a notable increase of obstetric haemorrhage and hypertensive disorders is noticeable. Contrary to such findings, incidence of venous thromboembolism has remained the same [2].

Set in the last decade of the previous century, Millennium Development Goals have led to unprecedented progress in terms of maternal mortality reduction to more equitable access of maternal health services and has resulted globally in maternal mortality reduction of nearly 50% between 1990 and 2015 [3]. Today, more than 20 years after the onset of the new millennium, we are faced with a new global threat, which has thwarted these ambitious goals set to provide ubiquitous welfare of motherhood. Instead of universal prosperity, the world has encountered a universal health disruptor, the SARS-CoV-2 infection. In the hearts and minds of the most vulnerable ones, which are our pregnant patients, COVID-19 started echoing as the greatest fear and the worst enemy. Across the globe, advanced maternal and gestational age, increased parity, obesity, and comorbidities have shown undoable association with poor maternal and pregnancy outcomes when complicated with the COVID-19 disease [4].

During the COVID-19 pandemic, Serbia faced devastating losses related to increased mortality rates among men and women of all ages [5]. As of March 2023, the total number of infected patients has reached more than 2.5 millions, with nearly 18,000 recorded cases of death [6]. Official data released by the Serbian health statistical yearbook revealed that the maternal mortality ratio per 100,000 live births has dramatically changed as well, from 6.2 in 2019, to 9.7 in 2021, culminating with 22.5 in 2021 [7]. Similar findings are coming from the other parts of the world as well. The United States reported in 2020 a maternal mortality incidence increases of more than 30% [8]. COVID-19 has led to disruption and exacerbation of previous, chronic medical conditions, or it was identified as a direct link leading to poor maternal outcomes, and therefore it is difficult to assess the real impact of the COVID-19 disease [8]. Unanimous conclusions regarding this dangerous disease were drown based on publications coming from numerous maternity units across Europe and over the globe, but the proportion of deaths associated directly to SARS-CoV-2, and the indirect consequences of COVID-19 pandemic have not been distinguished, get [9,10,11]. 

Studying the consequences of the COVID-19 pandemic on maternal outcomes is vivifying and stimulating for many professionals and decision-makers. Empirical evidence in obstetrics clinical practice has so far shown higher probability of adverse outcomes among pregnant women [12]. Wide variations and inconsistencies across the world in mortality rates associated with SARAS-CoV-2 have reported [13], and therefore any new evidence is a step forward in the fight against this disease. Furthermore, knowing the contextual characteristics can facilitate the application of literature findings in practice, which makes the exchange of experiences the most attractive and necessary to save lives and resources, especially if the evidence arises from the treatment of seriously ill pregnant women during the pandemic. Therefore, the aim of this study was to present findings of maternal mortality, as well as the risk factors associated with poor maternal outcome, among pregnant women infected with SARS-CoV-2 in Serbia.

## 2. Methods

### 2.1. Study Design

This was an observational, retrospective study of maternal mortality associated with the COVID-19 disease, and it included 192 subjects. All patients had a positive polymerase chain reaction (PCR) test for SARS-CoV-2 and they were hospitalized in the University hospital “Dr. Dragisa Misovic”, a tertiary level hospital in Belgrade, Republic of Serbia. The study lasted for more than a year and a half, starting in March 2020 and lasting until November 2021. It was approved by the hospital’s Institutional Review Board (decision no. 01-8816, August 2020), and it included all pregnant patients hospitalized during this timeframe. A negative PCR test for SARS-CoV-2 and multiple pregnancies were defined as exclusion criteria for the subject’s enrolment. All other hospitalized patients were enrolled in the study, and none of them was vaccinated. 

### 2.2. Study Participants

During COVID-19 outbreaks in 2020 and 2021, our hospital served as a referral centre for pregnant women facing a severe form of SARS-CoV-2 infection. Patients were referred to our hospital from the other maternity units and COVID-19 hospitals in Serbia due to the severity of their maternal condition caused by the infection. Admission criteria to our institution were defined according to an adapted version of the Modified Early Obstetric Warning Score (MEOWS) [14]. An intensive care unit (ICU) transfer was indicated in cases where it was not possible to maintain blood pressure values below 110 mmHg and 180 mmHg for diastolic and systolic blood pressure, respectively; if heart rate (HR) values were above 120 beats per minute; for oxygen blood saturation below 90%; a respiratory rate of less than 10 or higher than 30 breaths per minute; body temperature over 38 degrees Celsius; if a patient was unresponsive to treatment, and if there was a loss of verbal communication, consciousness, and disorientation. 

Due to the specificity of each pregnant patient’s individual needs, our patients were treated by multidisciplinary teams, according to national protocols for COVID-19 infection, issued by Serbia’s health authorities [15]. Accordingly, patients were treated with prophylactic doses of low-molecular-weight heparin (LMWH), while therapeutic doses were introduced in cases of suspected or confirmed venous thrombosis. Antibiotics were indicated in cases of a proven or at least likely bacterial superinfection, and corticosteroids (methylprednisolone) were recommended in order to prevent a cytokine storm in cases of severe maternal conditions, usually after the fourth day of disease onset.

Comparisons in this study were made between two groups of patients: a group of deceased and a group of hospital survivors with COVID-19.

### 2.3. Study Variables

Variables were divided in three study sets, with a total of 33 variables. 

The first set of variables included data related to symptoms and characteristics of infection: fever, dyspnea (difficult breathing), cough, fatigue (tiredness), headache, loss of taste and smell, diarrhoea, the number of days from symptom onset until hospitalization was initiated, and the pandemic wave. 

In Serbia, during 2020 and 2021, four outbreaks of diseases coincided with the dominance of four SARS-CoV-2 variants of concern (VOC): alpha, beta, gamma, and delta [16]. Based on the dynamics of our patients’ admission and discharge, the first pandemic wave, associated with the dominance of beta VOC, lasted from March until August 2020; the second pandemic wave was associated with the dominance of alpha VOC and it lasted from October until December 2020; gamma VOC was associated with the third pandemic wave and it lasted from February until May 2021; the fourth pandemic wave, associated with delta VOC, lasted from September until November 2021. 

The second set of variables included pregnancy-related features and comorbidities, including gestational age at the admission to the hospital, gestational hypertension, preeclampsia, gestational diabetes mellitus, and anemia. Maternal anthropometric characteristics were retrieved from primary health service reports, and they were used to calculate pre-pregnancy body mass index (BMI) values. Gestational hypertension and preeclampsia were defined according to the American College of Obstetricians’ and Gynaecologists’ (ACOG) guideline [17], and the American Diabetes Association’s (ADA) recommendations were used to define gestational diabetes mellitus [18].

Clinical endpoint datasets were collected from patients’ hospital medical records, and they were part of the third set of variables. These data contained mechanical ventilation requirement information, as well as the number of days that mechanical ventilation was required, the number of antibiotics administrated during the course of hospitalization, use of corticosteroids, the presence/absence of X-ray–confirmed pneumonia upon admission, nosocomial infections, pulmonary embolism, and abnormal uterine bleeding during delivery (postpartum haemorrhage). Pulmonary embolism diagnosis was established by YEARS diagnostic algorithm [19], and postpartum haemorrhage was defined as blood loss equal to or greater than 1000 mL, as well as blood loss accompanied by symptoms and signs of hypovolemia within 24 h following the delivery [20]. Depending on clinical and laboratory findings, Computed Tomography (CT) was performed in selected cases and lung involvement was assessed according to a chest CT score proposed by Li and associates [21].

### 2.4. Statistical Analysis

Results were presented as whole numbers (N) and percents (%) for categorical variables and as mean values (MV) and standard deviation (SD) for continuous variables. SPSS Statistics V.22.0. was used for all statistical tests. A Mann–Whitney test was used for comparisons between tested groups for the continuous variables, and for categorical variables, Chi square test and Fisher’s exact test were used. Statistical significance was set at *p* < 0.05.

## 3. Results

Out of 192 pregnant women who were treated in our hospital due to COVID-19 infection during pregnancy, a lethal outcome occurred in seven (3.6%) cases.

During the first wave of the pandemic, from March to the end of August 2020, the largest number of pregnant women with COVID-19 infection were hospitalized. In this period, not a single lethal outcome due to COVID-19 infection was recorded, Table 1. The fewest hospitalized pregnant patients with COVID-19 were recorded in the period from September 2020 to the end of January 2021. During that period, one pregnant woman died due to infection with the coronavirus. In the next two waves, hospital treatment for COVID-19 infection had approximately the same number of pregnant women. In the period from February to May 2021, a lethal outcome due to SARS-CoV-2 infection was recorded in two cases (28.6% of all subjects with a lethal outcome), while the highest number of deaths was recorded in the period from September to the end of November 2021. In that period, four (57.1% of all recorded lethal outcomes) pregnant women had a lethal outcome due to COVID-19 infection. Between the observed waves of the pandemic, age, and BMI values, no statistically significant difference was observed in the outcome of the infected pregnant women, Table 1.

**Figure 1:**
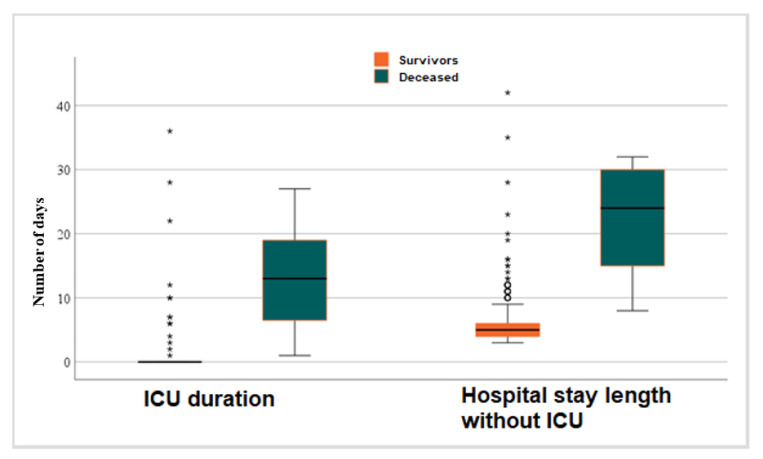
Length of hospitalization and ICU length and the outcome of treatment of pregnant women with COVID-19 infection (*n* = 192 patients; for ICU duration * *p* < 0.001; for hospital stay length without ICU * *p* < 0.001; *-Mann–Whitney test).

No statistically significant difference was observed in the duration of the disease in relation to the outcome of the treatment of pregnant women suffering from COVID-19 disease, Table 2. A statistically significant difference was observed in the number of days of hospitalization of deceased pregnant women and those who survived, Table 3. The women who died due to a COVID-19 infection had statistically significantly longer hospitalizations. The average length of a hospital stay of the subjects who survived was 6.39 + 5.13 days, and the average length of stay of deceased patients was 22.0 + 9.64 days, Figure 1.

The length of ICU duration statistically significantly differed between the subjects with a lethal outcome and those who survived, Table 2. Subjects with a lethal outcome were treated in the ICU for a statistically significantly longer time. The average length of treatment in the ICU was 0.86 + 4.04 days in the group of survivors, and 13.14 + 9.17 days in the group of deceased, Figure 1.

**Figure 2:**
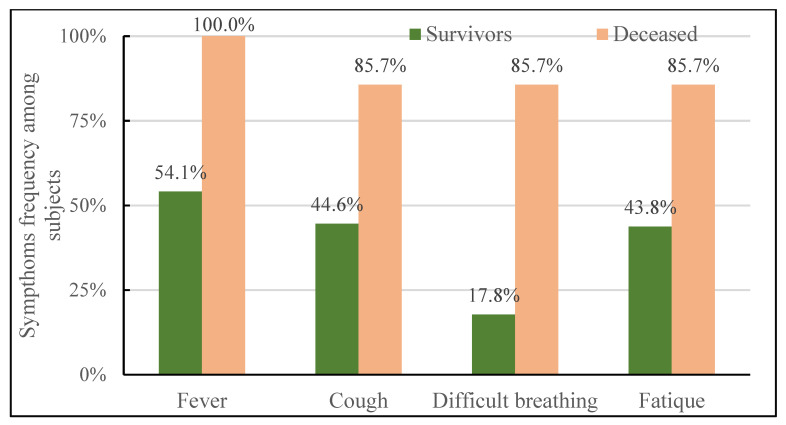
Differences in COVID-19 symptoms’ manifestation between the groups of pregnant patients.

The frequency of different symptoms of the COVID-19 infection among pregnant women are presented in Table 3. Between the subjects with a lethal outcome and those who survived, no statistically significant difference in frequency was observed in red or irritated eyes, sore throat, headache, loss of smell and taste, and diarrhoea, Table 3. In the group with a lethal outcome due to infection, there were statistically significantly more pregnant women with a body temperature of >38 °C on admission, cough, dyspnea, and fatigue, Figure 2.

Between the subjects with a lethal outcome and those who survived and were cured of the COVID-19 infection, a statistically significant difference was observed in the frequency of nosocomial infections, pulmonary embolism, postpartum haemorrhage, and the progression of the disease during the course of hospitalization, Table 4. 

Initial X-ray findings confirmed pneumonia in 33% of pregnant women with COVID-19 infection. A statistically significant difference was observed in the frequency of subjects with radiographically confirmed pneumonia upon admission between the two groups. An amount of 57.1% of pregnant women with a lethal outcome had X-ray confirmation of pneumonia upon admission, compared to 32.1% of pregnant women who survived, Table 5. An amount of 24.3% of the subjects who were cured of the COVID-19 infection and 71.4% of the subjects with a lethal outcome had CT diagnostics, Table 6. No statistically significant difference in the CT severity score was observed regarding the outcome of the treatment of the COVID-19 infection. In the group of cured patients, the average value of CT severity score was 10.42 + 6.75, and in the group with a lethal outcome, the average value of CT severity score was 14.40 + 2.61, Table 5.

No statistically significant differences were observed in the frequency of pregnant women who used antibiotics before the beginning of the hospitalization, in relation to the treatment outcome. The number of patients who required mechanical ventilation, duration of mechanical ventilation, average number of antibiotics administrated per patient, and the use of corticosteroids, statistically significantly differed between the two compared groups, Table 7.

In the deceased group, subjects were on average in their 28th gestational weak (192.14 ± 88.46 days), compared to the group of survivors who were on average in the 37th gestational week (258.23 ± 49.16 days). Aside from gestational age, statistically significant differences between compared groups were observed in the incidence of preeclampsia and gestational hypertension, Table 7. 

## 4. Discussion

Out of 192 subjects in this study, a lethal outcome occurred in seven cases. Deceased patients had longer hospitalization, number of days in ICU, were presenting more commonly at admission with X-ray–confirmed pneumonia, had a body temperature of >38 °C, and had coughing, dyspnea, and fatigue. They were more likely to have a progression of disease, to be dependent from mechanical ventilation, as well as to have nosocomial infection, pulmonary embolism, and postpartum haemorrhage. They were on average in their early third trimester of pregnancy, presenting more commonly with gestational hypertension and preeclampsia.

Eastern Europe and Southern Asia countries accomplished an exceptional maternal mortality reduction rate of 70% between 2000 and 2020 [22], due to significant efforts made in order to improve the healthcare system in these countries. With four registered cases of maternal death in 2019, Serbia, a country situated in Southeastern Europe, was closer than ever in reaching its long-term goals and joining the community of the leaders of maternal care in Europe [7,23]. In 2021, official, gloomy statistics dashed all our efforts, revealing 14 cases of maternal death [7] that happened in Serbia during the most difficult year of the COVID-19 pandemic for our healthcare system [24]. Half of all tragic outcomes reported in the country occurred exactly in our hospital, since all seven lethal outcomes in this study had happened during 2021, between January and November. 

A lethal outcome was more likely to occur during dominance of delta VOC, between September and November 2021. Due to its high contagiousness, delta VOC was speeding at a higher exponential rate than any other SARS-CoV-2 VOC, affecting particularly vulnerable groups of patients, and leading millions of people across the globe to death [25]. Although we had gained more experience and knowledge in our favour up to this point, delta VOC turned out to be superior to all our efforts. Shortly after its outbreak, maternity units across the world reported increased mortality rates associated with delta VOC dominance as well [26], and it became obvious that the pandemic was taking a new course, leading us into unknown agony. In 2021, we were very well averred of patients’ features that might direct us towards those who have a higher probability for a severe COVID-19 pregnancy outcome, as well as to distinguish between low- and high-risk patients [27]. Vaccination was taking its offensive course, but resistance and hesitation regarding its effectiveness and safety profiles took a toll [28]. New study results started appearing, such as the study conducted in the United States, which alerted the medical community that among unvaccinated patients, the delta variant was associated with more serious maternal mortality, compared to the pre-delta period [29]. Unfortunately, none of the patients in our study were vaccinated, and now, more than a year later, based on our own experiences and literature review, we can allay our doubts in favour of equal access to vaccination and to advocate COVID-19 vaccination in this vulnerable group of patients [30]. Blakeway et al. stressed the importance of better, clear communication with our patients as well as postvaccination surveillance, in order to reach our patients and overcome their hesitancy [31]. 

Compared to their non-pregnant counterparts, a mild form of COVID-19 disease is more common among pregnant women, but unfortunately, the risk of developing a severe form of SARS-CoV-2 infection is higher [32]. Therefore, triage and initial risk assessment remain a key link in our patient chain of care, as well as timely medical intervention and subsequent treatment [33]. Dyspnea, cough, fatigue, and body temperature of >38 °C were symptoms of infection leading more commonly to a critical form of infection and death. Unlike the other signs and symptoms of infection that we have collected and presented, these four clearly differed between two groups and, therefore, they must not be neglected on initial examinations and should be used for risk prediction. Other authors have warranted the importance of these symptoms of disease as well [34]. We have observed a significant difference in dyspnea manifestation: 85.7% of patients in the decedent group initially presented with this symptom, compared to 17.8% in the group of survivors. Pregnancy leads to changes in maternal respiratory functions and dyspnea is a common feature of normal pregnancy [35]. However, the onset of dyspnea should not be neglected and should be carefully analysed, since it may be caused by a pregnancy complication, such as venous thromboembolism and pulmonary embolism [34]. Even though dyspnea is the most common clinical feature of pulmonary embolism, a study conducted by Dresang et al. concluded that only four percent of clinically suspected cases of pulmonary embolism are actually confirmed in pregnant women [36]. Our experiences, on the other hand, imply a significant association between this clinical manifestation and severity of COVID-19 infection. She et al. argued the impact of fever and dyspnea on a poor COVID-19 outcome, and concluded that dyspnea, cough, fatigue, and fever are the most common clinical symptoms related to SARS-CoV-2 infection, but dyspnea was positively associated with mortality risk more than any other symptom [37]. 

The median length between COVID-19 symptoms’ onset and the initiation of hospitalization was approximately five days in both groups. In the study conducted by Pierce-Williams et al., the average duration of symptoms was seven days [38], a two-day difference compared to our study results, which may have led to a significant distinction in the morbidity and mortality outcome. Pierce-Williams et al. have not reported any case of maternal death [36], and therefore, this difference should be taken into consideration seriously and analysed thoroughly, since in the context of this parameter only, earlier initiation of hospitalization seems to be unbeneficial. Moreover, prolonged hospitalization is associated with the increased risk of hospital-acquired infections, especially with the late onset of infection [39], requiring strict infection control measurements and mandatory microbiological surveillance [40]. The decedent group of patients had nearly four times the number of prescribed antibiotics compared to the group of survivors, and more importantly, the incidence of nosocomial infections was significantly higher. More than half of all patients in the study group were treated with antibiotics before they were transferred to our institution, and the percentage share did not significantly differ between the two groups; it ranged from 53.5% to 57.1% in the favour of the decedent group. The average length of hospital stay and average ICU duration was nearly 16 and 12 days, respectively, and they clearly differed between the two groups. Prolonged hospitalization, and especially a longer ICU stay, is undoubtedly associated with the risk of co-infections, development of nosocomial infections, and the overall risk for a lethal outcome. Such conclusions are uniformly supported by other researchers’ experiences [41,42,43]. Pregnant women with COVID-19 disease are more likely to be admitted to the ICU and to require mechanical ventilation [27,44]. Disease progression led to critical illness, ICU admission, and mechanical ventilation requirements of our patients. All seven patients in the decedent group were dependent at some point on some mode of mechanical ventilation. When admitted to ICU, patients were treated in a multidisciplinary setting. ICU patients were treated by intensivists, constant obstetrical surveillance was provided, and depending on patients’ individual needs, consultations from other specialists were arranged. 

The incidence of hypertensive disorders during pregnancy (HDP) continues to increase worldwide, and despite all efforts, HDP remains the leading cause of maternal mortality [45]. Gestational hypertension was diagnosed in 23 cases, preeclampsia in 11, and their frequency of occurrence significantly differed between the groups. A strong association between gestational hypertension and preeclampsia with severity of COVID-19 disease among pregnant women is confirmed by meta-analysis [27], but reported incidences of 7.5% of a preeclampsia-like syndrome among women with severe COVID-19 disease convince us to remain cautious when diagnosing preeclampsia [46]. According to most researchers’ experiences, preexisting comorbidities are at the core of the cascade leading pregnant patients to poor outcomes [47,48], but our study results imply that pregnancy-related complications were the dominant ones and they seem to have played a key role in disease progression. A lack of diagnosis of chronic diseases, and the significant display of interindividual variability, puts us under an obligation to reevaluate the facts, as well as to distinguish the real impact of SARS-CoV-2 infection and the indirect consequences of the disease triggered by the complex set of interactions between the angiogenic imbalance and endothelial damage [49]. Advanced maternal age and obesity are very well recognised risk factors for poor maternal outcomes [45]. Patients in the decedent group were on average two years older and had higher BMI values, but the significant difference was not established. A lack of statistically significant differences between these two parameters, as well as the lack of diagnoses of preexisting comorbidities, leads us to the assumption that the interception of some other factors, such as genetic or immunological, could have played a more prominent role in the outcome prediction [50,51]. Gestational age seems to have been another deteriorating risk factor for poor maternal outcome; patients in the decedent group were in their early third trimester, contrary to the group of survivors who were in their middle or late third trimester of pregnancy. The third trimester of pregnancy is associated with the highest susceptibility for COVID-19 infection [27,52]. Observed differences in disease onset might imply that pregnant women are in greater jeopardy during their early third trimester and might alarm obstetricians to be more cautious when treating patients in this gestational age. 

This study has several limitations. The sample size of the deceased group was small and did not permit reliable comparisons with the sample of survivors. However, the found statistical significance should not be disregarded even in small samples, as per the medical ethics of saving lives and effective use of public health resources. Furthermore, even though the simple statistical analyses do not permit robust conclusions, it first shows what is possible with limited datasets, often only available in low and middle-income countries. It points to a need to encourage other patient data collection during the pandemic. Lack of knowledge regarding the study population’s size is certainly another limitation in this study, and therefore, the representativeness of our findings is limited. Furthermore, study results cannot be generalised to other institutions and populations where different therapeutic protocols for COVID-19 disease were applied, despite their validity. 

## 5. Conclusions

Pregnant women facing COVID-19 disease should be carefully and thoroughly analysed when presenting at our practices and hospitals. Initial clinical manifestations of SARS-CoV-2 infection, such as dyspnea, cough, fatigue, and fever, could be potent factors in risk stratification and outcome prediction. Prolonged hospitalization, ICU admission, and associated risk of hospital-acquired infections require strict microbiological surveillance and should be a constant reminder of rational antibiotics use.

Identification and understanding of risk factors associated with poor maternal outcomes among pregnant women infected with SARS-CoV-2 should warn medical professionals of potentially unwanted outcomes and can be used for organising an individualised treatment for a pregnant patient’s specific needs, including a guide to necessary consultations with medical specialists in various fields. Each death of a pregnant women remains a wound that does not heal, and it inevitably leads us to reflect on the actions and choices we have made in order to save a life, carrying a new life at the same. Constant reevaluation of facts and circumstances that lead to poor outcomes as well as continual research should be pursued and must not be neglected in the upcoming years.

## Figures and Tables

**Table 1:** Distribution of participants in each pandemic wave and anthropometric parameters of pregnant women with COVID-19 disease regarding outcome.

Study Variables X ± SD/*n* (%)		Total Number of Subjects	Comparisons between Two Groups of Subjects Infected with COVID-19		Statistical Significance
			Survivors	Deceased	
**Pandemic wave**	**Wave 1** **(beta VOC)**	61 (31.8%)	61 (33.0%)	0 (0%)	^a^ *p* = 0.121
	**Wave 2** **(alpha VOC)**	38 (19.8%)	37 (20.0%)	1 (14.3%)	
	**Wave 3** **(gamma VOC)**	47 (24.5%)	45 (24.3%)	2 (28.6%)	
	**Wave 4** **(delta VOC)**	46 (23.9%)	42 (22.7%)	4 (57.1%)	
**Age (years)**		30.69 ± 5.34	30.63 ± 5.39	32.29 ± 3.68	^b^ *p* = 0.421
**Body mass Index (BMI)**		27.16 ± 3.70	27.10 ± 3.46	28.62 ± 8.13	^c^ *p* = 0.265

^a^ χ^2^-test; ^b^ *t* test; ^c^ Mann–Whitney test; VOC—variant of concern.

**Table 2:** Duration of COVID-19 symptoms before the onset of hospitalization, length of hospitalization, and ICU duration regarding outcome.

Study Variables X ± SD	Total Number of Subjects	Comparisons between Two Groups of Subjects Infected with COVID-19		Statistical Significance
		Survivors	Deceased	
**Days from COVID-19 symptom onset to hospital initiation (days)**	4.72 ± 4.67	4.71 ± 4.74	5.0 ± 1.63	^a^ *p* = 0.268
**Hospital stay length without ICU (days)**	6.96 ± 6.07	6.39 ± 5.13	22.0 ± 9.64	^a^ *p* < 0.001 *
**ICU duration (days)**	1.31 ± 4.87	0.86 ± 4.04	13.14 ± 9.17	^a^ *p* < 0.001 *

* statistically significant difference; ^a^ Mann–Whitney test.

**Table 3:** Symptoms of COVID-19 infection recorded on the hospital admission regarding outcome.

Study Variables *n* (%)	Total Number of Subjects	Comparisons between Two Groups of Subjects Infected with COVID-19		Statistical Significance
		Survivors	Deceased	
**Red or irritated eyes**	7 (3.6%)	7 (3.8%)	0 (0%)	^a^ *p* = 0.600
**Sore throat**	40 (20.8%)	39 (21.1%)	1 (14.3%)	^a^ *p* = 0.664
**Body temperature > 38 °C on admission**	107 (55.7%)	100 (54.1%)	7 (100%)	^a^ *p* = 0.016 *
**Cough**	88 (46.1%)	82 (44.6%)	6 (85.7%)	^a^ *p* = 0.036 *
**Dyspnea**	39 (20.3%)	33 (17.8%)	6 (85.7%)	^a^ *p* < 0.001 *
**Headache**	28 (14.6%)	26 (14.1%)	2 (28.6%)	^a^ *p* = 0.285
**Smell lost**	57 (29.7%)	54 (29.2%)	3 (42.9%)	^a^ *p* = 0.437
**Taste loss**	53 (27.6%)	50 (27.0%)	3 (42.9%)	^a^ *p* = 0.358
**Fatigue**	87 (45.3%)	81 (43.8%)	6 (85.7%)	^a^ *p* = 0.029 *
**Diarrhoea**	6 (3.1%)	6 (3.2%)	0 (0%)	^a^ *p* = 0.628

* statistically significant difference; ^a^ χ^2^-test.

**Table 4:** Clinical endpoints distribution between pregnant women who survived COVID-19 disease and deceased pregnant women.

Study Variables *n* (%)	Total Number of Subjects	Comparisons between Two Groups of Subjects Infected with COVID-19		Statistical Significance
		Survivors	Deceased	
Clinical endpoints				
**Nosocomial infection**	20 (10.4%)	13 (7.0%)	7 (100%)	^a^ *p* < 0.001 *
**Pulmonary embolism**	2 (1.1%)	1 (0.6%)	1 (14.3%)	^a^ *p* < 0.001 *
**Postpartum haemorrhage**	3 (1.6%)	1 (0.5%)	2 (28.6%)	^a^ *p* < 0.001 *
**Progression of COVID-19 disease**	32 (16.8%)	25 (13.6%)	7 (100%)	^a^ *p* < 0.001 *

* statistically significant difference; ^a^ χ^2^-test.

**Table 5:** Distribution of diagnostic imaging modes and CT severity score in tested patients regarding outcome.

Study Variables X ± SD/*n* (%)	Total Number of Subjects	Comparisons between Two Groups of Subjects Infected with COVID-19		Statistical Significance
		Survivors	Deceased	
**Computed tomography (CT) performed**	50 (26.0%)	45 (24.3%)	5 (71.4%)	^a^ *p* = 0.600
**CT severity score**	10.82 ± 6.56	10.42 ± 6.75	14.40 ± 2.61	^b^ *p* = 0.087
**X-ray confirmed pneumonia upon hospital admission**	63 (33.0%)	59 (32.1%)	4 (57.1%)	^a^ *p* < 0.001 *

* statistically significant difference; ^a^ χ^2^-test; ^b^ Mann–Whitney test.

**Table 6:** Applied therapeutic measurements and COVID-19 disease outcomes.

Study Variables X ± SD/*n* (%)	Total Number of Subjects	Comparisons between Two Groups of Subjects Infected with COVID-19		Statistical Significance
		Survivors	Deceased	
**Patients requiring mechanical ventilation**	35 (18.2%)	28 (15.1%)	7 (100%)	^a^ *p* < 0.001 *
**Duration of mechanical ventilation (days)**	2.03 ± 5.83	1.42 ± 4.71	17.86 ± 9.56	^b^ *p* < 0.001 *
**Use of antibiotics before the hospitalization**	103 (2.6%)	99 (53.5%)	4 (57.1%)	^a^ *p* = 0.850
**Average number of antibiotics administrated per patient**	1.72 ± 1.42	1.57 ± 1.19	5.71 ± 1.25	^b^ *p* < 0.001 *
**Corticosteroids use**	25 (13.00%)	18 (9.7%)	7 (100%)	^a^ *p* < 0.001 *

* statistically significant difference; ^a^ χ^2^-test; ^b^ Mann–Whitney test.

**Table 7:** Pregnancy-related features in study participants regarding outcome.

Study Variables X ± SD/*n* (%)	Comparisons between Two Groups of Subjects Infected with COVID-19		Statistical Significance
	Survivors	Deceased	
Pregnancy related feature			
**Gestational age (days)**	258.23 ± 49.16	192.14 ± 88.46	^a^ *p* < 0.001 *
**Gestational hypertension**	19 (10.3%)	3 (42.9%)	^b^ *p* < 0.034 *
**Preeclampsia**	8 (4.3%)	3 (42.9%)	^b^ *p* = 0.004 *
**Gestational diabetes mellitus**	14 (7.6%)	0 (0.0%)	^b^ *p* = 1.000
**Anemia**	67 (36.2%)	4 (57.1%)	^b^ *p* = 0.427

* statistically significant difference; ^a^ Mann–Whitney test; ^b^ Fisher’s Exact test.

## Data Availability

All data are presented in this study. Original data are available upon reasonable request from the corresponding author.
